# Patterns of spinal motion, kinematic spaces and the land‐to‐sea transition in carnivorans

**DOI:** 10.1002/ar.70102

**Published:** 2025-12-24

**Authors:** Juan Miguel Esteban, Alberto Martín‐Serra, Alejandro Pérez‐Ramos, Borja Figueirido

**Affiliations:** ^1^ Departamento de Ecología y Geología, Facultad de Ciencias Universidad de Málaga Málaga Spain

**Keywords:** aquatic adaptations, fissipeds, mobility, pinnipeds, vertebrae

## Abstract

The vertebral column of pinnipeds (seals and kin) has undergone profound morphological changes, yet the functional consequences of these changes remain poorly explored. Here, we quantify the osteological range of motion (*oROM*) of presacral intervertebral joints across the cervical, thoracic, and lumbar regions of pinnipeds, as well as terrestrial and semi‐aquatic carnivores for comparative purposes. To do this, we used 3D reconstructions of vertebral columns, and we applied *Autobend*, an experimentally validated methodology to quantify intervertebral joint mobility across axial, lateral, and sagittal planes from dry vertebrae. Our results reveal that pinnipeds exhibit greater intervertebral mobility than non‐aquatic taxa, particularly in the lumbar region, which probably relates to their aquatic adaptations. However, cervical mobility is more reduced in pinnipeds, likely due to decreased reliance on head maneuverability. We also demonstrate differences among pinniped families that may relate to their distinct swimming styles and locomotion on land. Accordingly, the spine of otariids retains higher flexibility, while that of phocids exhibits greater coordination and rigidity across the thoracic region but high mobility in the lumbar spine. Odobenids show a unique pattern with reduced cervical mobility but high thoracic and lumbar flexibility. Finally, we relate these kinematic patterns to previously published data on vertebral integration and modularity. Our results may agree with previous hypotheses that reduced morphological integration is associated with increased mobility and vice versa.

## Graphical abstract

Using 3D reconstructions, we quantify intervertebral joint mobility to investigate how cervical, thoracic, and lumbar vertebral regions evolved distinct kinematic roles in pinnipeds. The findings reveal lineage‐specific adaptations for swimming and highlight the functional significance of vertebral joint flexibility in ecological transitions.

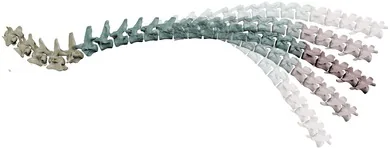

## 1 INTRODUCTION

The vertebral column is a key structure of the mammalian body plan because it protects the spinal cord, maintains body posture, and facilitates locomotion (Gál, 1993; Galbusera & Bassani, 2019; Jones et al. 2018; Slijper, 1946). In addition, in land‐dwelling mammals, the presacral spine aids in withstanding body weight against gravity and enabling force transmission from the limbs (Rockwell et al., 1938; Slijper, 1946). Aquatic taxa, however, have undergone a profound body plan reorganization in response to the “land‐to‐sea” macroevolutionary transition, including a shift from limb‐powered to column‐driven locomotion (Figueirido et al., 2022; Pierce et al., 2011; Thewissen & Fish, 1997). Indeed, recent studies have demonstrated changes in morphology, disparity, and modularity of the presacral column across different groups of mammals in response to this re‐invasion of the marine realm (e.g., Esteban et al., 2023a, 2023b, 2024; Gillet et al. 2024). Among them, pinnipeds (seals and kin) are carnivorans that evolved specialized traits to forage in the aquatic environment, including a streamlined (fusiform) body, paddle‐like limbs, and reduced external structures such as external ear pinnae and tails (e.g., Berta et al., 2006; Fish 1998). However, despite their aquatic adaptations, they still retain some degree of terrestrial habits for key behaviors such as mating and giving birth (Mittermeier & Wilson, 2014).

Esteban et al. (2024) investigated how the shift from limb‐powered to column‐driven locomotion and the release of the presacral column from its weight‐bearing function influenced vertebral column disparity across the evolution of pinnipeds. They demonstrated that stem pinnipeds exhibited novel morphologies not seen in modern forms, especially across the cervical and lumbar series. This suggests that the early semi‐aquatic transition of stem pinnipeds was characterized by an evolutionary scenario with a low morphological constraint. However, during the Miocene, pinnipeds evolved toward a more restricted (constrained) scenario concomitant to the diversification of the crown group.

Esteban et al. (2023a) compared morphological integration and modularity patterns of the presacral column in pinnipeds with those already obtained for terrestrial carnivorans by Martín‐Serra et al. (2021). The study showed that vertebral integration in pinnipeds was reduced compared with the one observed in their terrestrial relatives, with the implication of a modular reorganization along the column.

Regarding patterns of modularity, Martín‐Serra et al. (2021) proposed that the thoracolumbar column of terrestrial carnivorans is composed of the presence of two modules—sets of vertebrae that are more integrated among them than with other vertebrae. An anterodorsal module, which is composed of vertebrae with vertebrosternal ribs, is thought to provide trunk stability during locomotion. The second is the posterodorsal module, composed of thoracics with floating ribs plus the lumbar series, which is proposed to restrict the excessive movement generated in the posterior lumbar region when locomoting on land. Moreover, the diaphragmatic vertebra (i.e., the thoracic vertebra that shifts the orientation of pre‐ and postzygapophyses) is not integrated into either of the modules, and Martín‐Serra et al. (2021) and Figueirido et al. (2023) proposed that this vertebra may act as a hinge within both dorsal modules.

This pattern of vertebral column integration in terrestrial carnivorans is not present in pinnipeds. Indeed, Esteban et al. (2023a) demonstrated that the vertebral column of otariids does not appear to have a clear modular organization, whereas that of phocids possesses a distinct thoracic module composed of a highly integrated group of thoracic vertebrae (all those with vertebrosternal and floating ribs). Moreover, Esteban et al. (2023a) argued that these differences between otariids and phocids should be related to their different swimming strategies (Fish, 2016).

Phocids are hindlimb‐dominated swimmers that use pelvic oscillation—that is, the anterior body is held rigid, while lateral undulations of the lumbar region are coupled with lateral sweeps of the hind flippers to generate thrust (Fish, 1996; Fish et al. 1988; Hocking et al. 2021; Tarasoff et al. 1972). On land, phocids move like caterpillars, with the body in contact with the land and without the limbs supporting their body weight (Garrett & Fish, 2015; Kuhn & Frey, 2012). However, otariids are considered forelimb‐dominated swimmers, as they use their fore flippers to generate thrust (i.e., pectoral rowing) and the hindlimbs and axial skeleton play no apparent role in generating propulsion (e.g., Beentjes, 1990; English, 1976; Feldkamp, 1987; Fish, 1996; Hocking et al. 2021), but their axial skeleton seems very flexible for rapid maneuverability and turning (Fish et al., 2003; Leahy et al., 2021; Pierce et al., 2011). On land, they are able to walk using quadrupedal gaits (Kuhn & Frey, 2012). Finally, the only living odobenid, the walrus (*Odobenus rosmarus*), can perform the two types of locomotion deployed by phocids and otariids. The walrus swims primarily using pelvic oscillation, in a way similar to phocids, but it can also paddle with the forelimbs when moving at slow speeds (Gordon, 1981). In contrast, their land locomotion is like that of otariids (Gordon, 1981, 1983).

The studies of Esteban et al. (2023a) and Esteban et al. (2024) suggest that the novel morphologies of aquatic carnivorans are mainly driven by the “quasi‐relaxation” of terrestrial constraints and the emergence of new locomotory demands in the aquatic environment. However, while both studies provide important aspects of the evolution of vertebral shape and spinal integration concomitant to the land‐to‐sea transition, they do not directly address the functional implications of these changes. Therefore, it remains unknown if these changes in vertebral shape and spinal organization have direct consequences for the degree of flexion and extension across movement planes, an essential determinant of kinematic performance and locomotor capability (e.g., Belyaev et al., 2024).

Here, we quantify intervertebral joint mobility on the presacral spine of pinnipeds across different planes to investigate whether shifts in both vertebral shape and spinal organization relate to function across a major ecological transition. To do this, we calculate the osteological range of motion (*oROM*) of pairs of consecutive vertebrae using *Autobend* (Jones et al., 2024; Jones, Brocklehurst, & Pierce, 2021). This approach allows us to investigate whether certain spinal regions or pinniped groups exhibit increased or reduced spinal mobility and how this relates to swimming modes, terrestrial habits, or different feeding behaviors. Moreover, in this study, we also investigate the degree of coordinated mobility across different planes of motion. Movements in the sagittal, axial, and lateral planes may be highly coordinated or may vary independently. In functional terms, a high coordination is expected to be associated with stabilized or unified spinal motion. Conversely, a low coordination may be interpreted as allowing different parts of the spine to move semi‐independently. These different patterns among movement coordination can provide important clues into lineage‐specific functional adaptations of the vertebral column.

Therefore, the specific goals of this study are: (1) to quantify osteological range of motion (*oROM*) as a proxy for intervertebral joint mobility in the cervical, thoracic, and lumbar regions (Figure 1a) across five carnivoran groups (terrestrial carnivorans, semiaquatic carnivorans, phocids, otariids, and odobenids); (2) to compare the mobility values obtained across different spatial planes (i.e., axial, lateral, and sagittal) (Figure 1b) with the goal to assess whether non‐pinniped taxa (i.e., terrestrial and semiaquatic) markedly differ in regional movements from pinnipeds; (3) to explore whether there are regional differences in vertebral column mobility associated with the different locomotor strategies deployed by pinnipeds (i.e., pectoral rowing of otariids, pelvic oscillation of phocids, and the mixed‐type locomotion of odobenids). This also includes investigating correlations among motion planes to identify lineage‐specific strategies of movement coordination; and (4) to qualitatively compare the regional mobility patterns obtained here with previous findings on vertebral shape and column integration to better understand the functional implications of these changes during the land‐to‐sea transition of carnivorans.

**FIGURE 1:**
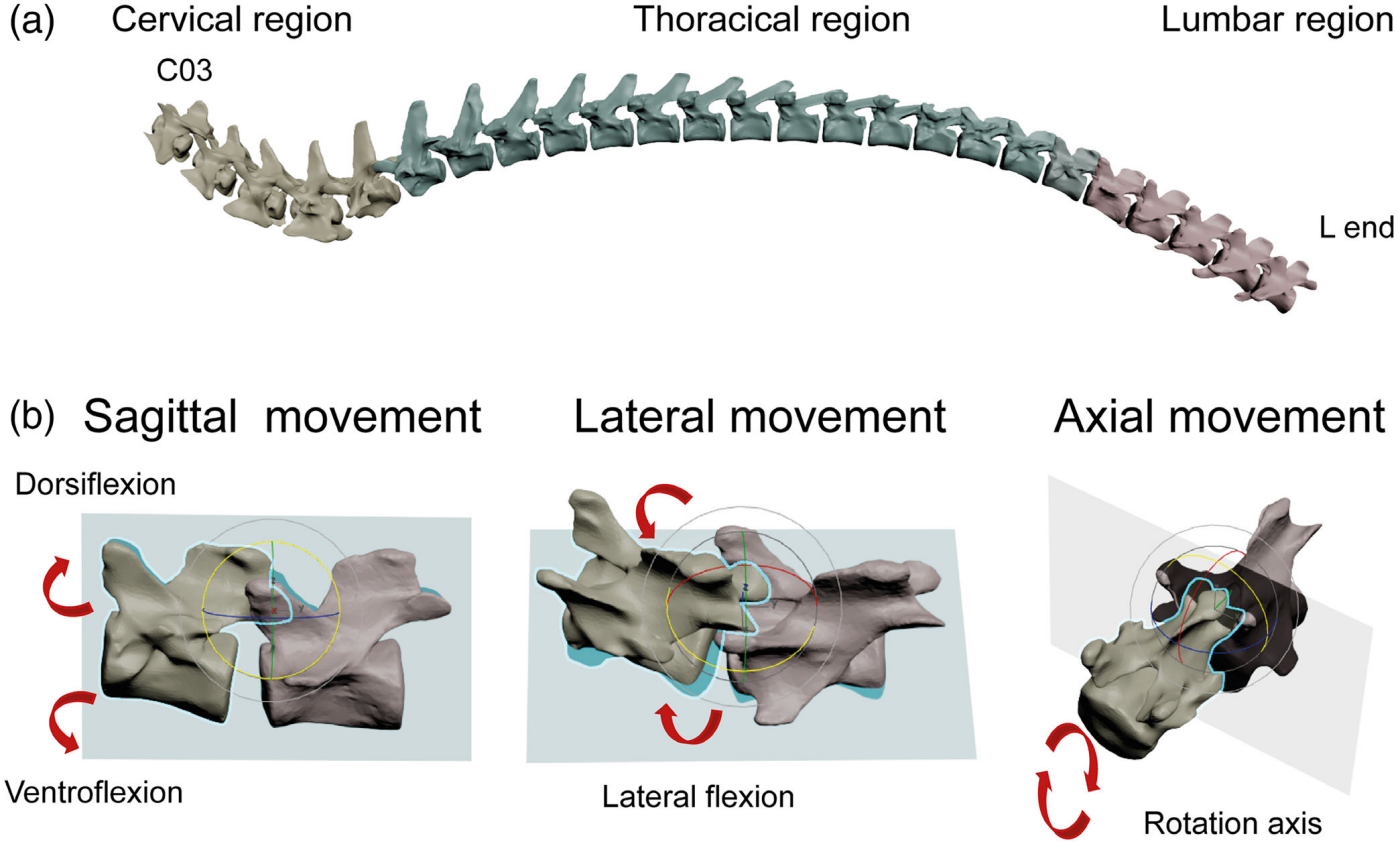
Vertebral column regions, planes of motion and movements to be quantified in this article. Presacral regions (a) and planes of motion (b) to calculate *oROM*.

## 2 MATERIALS AND METHODS

### 2.1 3D scanning

The osteological range of motion of intervertebral joints was calculated in 23 species, including 16 pinnipeds and seven species of musteloid carnivorans (6 mustelids and 1 procyonid, including terrestrial and semi‐aquatic species) (Table 1). All sampled specimens were adults.

**TABLE 1:** Sampled species and specimens used to conduct *oROM* analysis together with museum IDs, ecological habits, families to which each species belong, and institutions where the skeletons are housed.

Specie	ID	Ecology	Family	Institution
*Enhydra lutris*	N/A	Semiaquatic	Mustelidae	LACM
*Lutra canadiensis*	NUF 342	Semiaquatic	Mustelidae	CMN
*Lutra lutra*	2981	Semiaquatic	Mustelidae	UVA
*Martes foina*	7432	Terrestrial	Mustelidae	UVA
*Meles meles*	N/A	Terrestrial	Mustelidae	UVA
*Procyon lotor*	4264	Terrestrial	Procyoniae	UVA
*Odobenus rosmarus*	150	Aquatic	Odobenidae	ZMUC
*Odobenus rosmarus*	N/A	Aquatic	Odobenidae	NHMB
*Eumetopias jubatus*	N/A	Aquatic	Otariidae	MNHN
*Neophoca cinerea*	838	Aquatic	Otariidae	ZMUC
*Otaria byronia*	854	Aquatic	Otariidae	ZMUC
*Callorhinus ursinus*	1878.198	Aquatic	Otariidae	MNHN
*Zalophus woellibaeki*	63,957	Aquatic	Otariidae	AMNH
*Zalophus californianus*	263	Aquatic	Otariidae	ZMUC
*Arctocephalus pusillus*	N/A	Aquatic	Otariidae	AMNH
*Cystophora cristata*	1134	Aquatic	Phocidae	ZMUC
*Monachus tropicalis*	10,431	Aquatic	Phocidae	AMNH
*Halichoerus grypus*	975	Aquatic	Phocidae	ZMUC
*Mirounga angustirostris*	839	Aquatic	Phocidae	ZMUC
*Hydrurga leptonyx*	1970.325	Aquatic	Phocidae	MNHN
*Pagophilus groendlandicus*	782	Aquatic	Phocidae	ZMUC
*Phoca vitulina*	1077	Aquatic	Phocidae	ZMUC
*Erignathus barbatus*	958	Aquatic	Phocidae	ZMUC

*Note*: For the vertebral formulae and location of the diaphragmatic vertebrae, see Table S1.

Abbreviations: AMNH, American Museum of Natural History (New York, USA); CMN, Canadian Museum of Nature (Ottawa, Canada); LACM, Los Angeles County Museum (Los Angeles, California); MNHN, Museum nationalle d'Historie naturelle (Paris, France); NHMB, Naturhistorischen Museum Basel (Basel, Switzerland); UVA, Museo Anatomico de la Universidad de Valladolid (Valladolid, España); ZMUC, Zoological Museum, University of Copenhagen (Copenhagen, Denmark).

Each joint was assigned to a specific region based on the adjacent vertebrae. The articulation between the last vertebra of one region and the first vertebra of the subsequent region was classified as belonging to the most anterior one. Although different taxa exhibit variation in the number of thoracic and lumbar vertebrae, the total thoracolumbar count remains almost invariably at 20 vertebrae, except for *Odobenus rosmarus*, *Cystophora cristata*, and *Martes foina*, which possess 19, and *Mirounga angustirostris*, which possesses 21 (Table S1). The diaphragmatic vertebra was defined according to the orientation of zygapophyses. The exact position of the diaphragmatic vertebra varies across species and is shown also in Table S1.

All presacral vertebrae were scanned with an EinScan Pro Plus 2X surface scanner (resolution = 0.05 mm) to create three‐dimensional meshes. In some cases, minor surface data loss (e.g., small holes) required virtual repairs, which were performed using Geomagic Essentials (3D Systems, North Carolina, USA). Prior to assembling the 3D vertebral column, each mesh underwent polygon reduction to approximately 20,000 triangles to optimize computational efficiency for subsequent analyses while maintaining decimation to the original boundary of the mesh (Pérez‐Ramos & Figueirido, 2020).

### 2.2 Vertebral column assembly

The vertebral column was assembled in a neutral position using Geomagic Essentials (3D Systems, North Carolina, USA), following the protocol described by Jones, Brocklehurst, & Pierce (2021). The first two cervicals (atlas and axis) were not included due to their distinct morphology and articulation surfaces. The process involved: (i) importing vertebral meshes for accurate alignment; (ii) using the “N‐point alignment” tool, where three reference points were placed on the posterior surface of one vertebra and matched to three corresponding points on the anterior surface of the subsequent vertebra. This aligned the second vertebra relative to the first; and (iii) fine‐tuning the orientation of the posterior vertebra using the “Move: Advanced” tool, which provides translation and rotation along three axes. This step ensured that the central facets and zygapophyses were parallel and maximally overlapped (Figure 2a).

**FIGURE 2:**
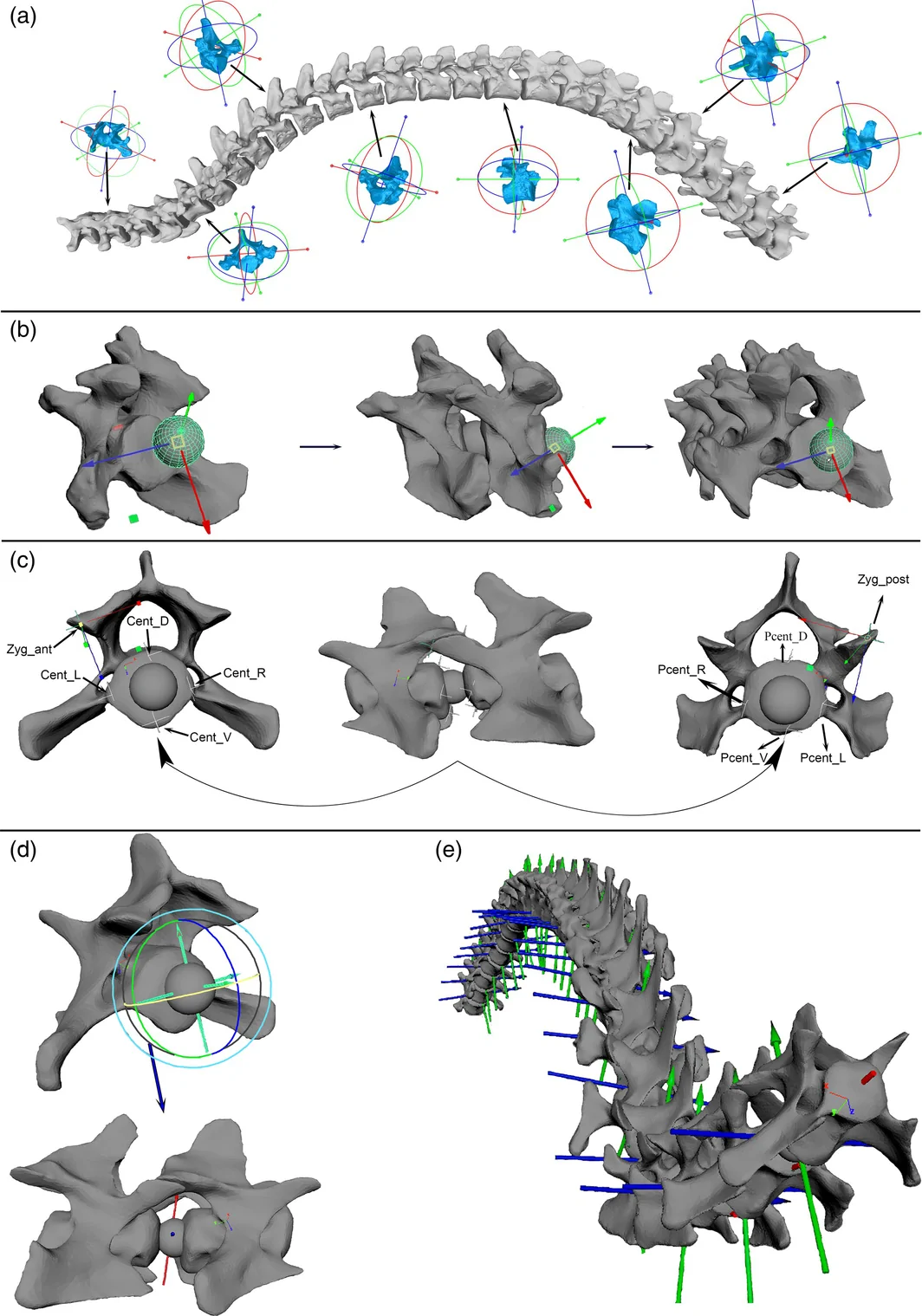
Quantification of *oROM*. (a) The assembly of the column is to be conducted in Geomagic Essentials, with the utilization of the ‘transform’ tool to facilitate the movement and assembly of the vertebrae. Blue vertebrae are taken as an example of orientation process. (b) The subsequent step involves the placement of the core (COR) between the anterior and posterior facet of the joint, which is to be carried out in Autodesk Maya. (c) The next stage is to place the 10 markers on each vertebra that constitutes the joint, with five markers on each vertebra. (d) The placement and preparation of the axes in the center of the COR is the final stage of the procedure. (e) The final view of an assembled spine is ready to analyze the *oROM*.

### 2.3 oROM quantification and analysis

Once assembled (Figure 2a), the vertebral column was exported as a .obj file and imported into *Autodesk Maya* for further analysis, using *AutoBend* (Jones, Brocklehurst, & Pierce, 2021). Following the established protocol: (i) the center of rotation (COR) for each intervertebral joint was set at the center of the intervertebral joint, aligned with the midpoints of the two centra (Figure 2b); (ii) ten reference points were placed at each joint to account for the impact of soft tissues during motion estimation (Figure 2c)—that is, they were placed to track intervertebral disc and zygapophyseal joint strain; (iii) motion was quantified along three axes: X‐axis (axial rotation), Y‐axis (lateral flexion), and Z‐axis (sagittal flexion) (Figure 2d); and (iv) *AutoBend* incorporates movement restrictions based on bone intersection while considering soft tissue constraints from the zygapophyses and centrum (Jones, Brocklehurst, & Pierce, 2021).

The movement restrictions validated by *AutoBend* (Jones, Brocklehurst, & Pierce, 2021) were analyzed using the following criteria (Figure 2): (i) bone intersection: overlap between the anterior and posterior vertebral meshes was measured using a Boolean object. Any overlap served as a stopping criterion for movement, though a small intersection threshold was permitted before cessation; (ii) zygapophyseal tension: soft tissue limitations in the synovial joint were assessed by analyzing the relative positions of the two zygapophyseal reference points; and (iii) centrum tension: soft tissue constraints within the intervertebral disc were evaluated based on the relative positions of eight centrum reference points.

*AutoBend* allows for the adjustment of permissibility parameters to accommodate different vertebral columns. The key parameters modified in this study include: (i) joint spacing, which represents the relative separation between two vertebrae due to soft tissues and cannot be precisely determined from bone data alone (Figure 2). To account for potential inaccuracies in model reconstruction, values were varied within a ± 10% range; (ii) the intersection threshold, which defines the permissible overlap between vertebrae during flexion movements. This parameter accounts for potential errors in 3D reconstruction, such as mesh inflation during wrapping or surface irregularities. The threshold was set between 0.25% and 0.5%; and (iii) tension, which refers to the location of the reference points used to estimate soft tissue displacements in the model. Misplacement of these points can lead to measurement inaccuracies in zygapophyseal and centrum tension. To mitigate this, tension values were adjusted within a range of −45% to +45%, allowing for minor variations in displacement estimations.

Each vertebral column was analyzed a total of eight times, varying the described parameters (i.e., joint spacing, intersection threshold, and location tension) in each analysis. The eight parameter combinations were generated by systematically combining the two values of joint spacing (−10%, +10%), the two of intersection threshold (0.25%, 0.5%), and the two of tension (−45%, +45%). This resulted in the following combinations: −10/0.25/−45, −10/0.25/+45, −10/0.5/−45, −10/0.5/+45, +10/0.25/−45, +10/0.25/+45, +10/0.5/−45, +10%/0.5%/+45%.

*AutoBend* provides six measurements in each joint: two measurements of torsion (left and right), two measurements of lateral flexion (left and right), one measurement of dorsal flexion, and one measurement of ventral flexion. The two measurements of each plane for the axial and lateral planes were summed to obtain the total range of motion in each of these planes. For the sagittal plane, we separated ventroflexion from dorsiflexion because they are not symmetrical and can show functional differences.

For each vertebral column, we averaged the values obtained for each joint across the eight parameter combinations, treating each axis of movement separately. This procedure follows the protocol established by Jones, Brocklehurst, & Pierce (2021), which was previously validated against ex vivo cadaveric data and yielded reliable estimates of intervertebral mobility.

### 2.4 Statistical analyses

We performed three Principal Component Analyses (PCA) from *oROM* values for each joint, considering the cervical, thoracic, and lumbar regions separately. Our main goal was to construct ‘kinematic spaces’ for the presacral spine, thereby identifying the principal axes of functional variation and distinguishing taxonomic/locomotory groups according to their mobility patterns. In addition, we calculated the mean of the scores obtained for each joint per group to describe the main patterns of kinematic variation at the group level. Group‐level averaging is essential to reveal general patterns, especially in our dataset that contains large numbers of joints from adjacent vertebrae, where variability at the individual level can obscure biologically meaningful trends.

To test whether groups defined by taxonomic and locomotor strategies exhibit distinct movement patterns—that is, whether they occupy different regions of the PCA morphospa—we conducted PERMANOVA tests separately for the cervical, thoracic, and lumbar regions. Each analysis was performed on the first two principal components of the respective PCA at individual‐level data, using the variable of group (non‐pinniped carnivorans, otariids, phocids, and odobenids) as a factor, implemented with the function PERMANOVA in the R environment (Anderson, 2005). This approach was adopted for the following reasons: (1) in all three PCAs, the first two components together explain over 75% of the total shape variance, ensuring that we capture most biologically relevant variation (see results); (2) limiting the analysis to these components also reduces the risk of incorporating noise from other PCs with negligible explanatory power; and (3) as PCs are orthogonal eigenvectors, the full independence among variables used is ensured.

To identify lineage‐specific strategies of movement coordination across the presacral column, we perform correlation analyses among movement planes with the data averaged per group. Specifically, we calculated the mean of *oROM* values per joint across all individuals in each group. The peculiarity of our dataset is that not all species of a given group possess the same number of thoracic and lumbar vertebrae (see Table S1). To address this issue, the boundary joints were ascribed to each region following the most frequent allocation in each group. For example, within otariids, six species possess 15 thoracics (joints 6–20) and five lumbars (joints 21–24) but one species possesses 16 thoracics (6–21) and four lumbars (22–24). Therefore, although the joint 21 is consistently averaged per group, it is analyzed with the thoracics because for most of the otariids this joint is a thoracic one. This might introduce a slight analytical bias in the correlation analyses, but this approach has the power to identify biologically meaningful coordination patterns along the column itself, rather than variation due to interspecific differences across species.

The correlation analyses were performed with the function cor() in R (R Core Team, 2023), and the correlation matrix was visually represented by a heatmap using the package corrplot (Wei et al., 2017).

## 3 RESULTS

### 3.1 Mobility profiles of the vertebral column across planes

The *oROM* analysis revealed variations in intervertebral joint mobility among the studied groups across the three spatial planes—axial, lateral, and the two components of sagittal mobility (i.e., dorsiflexion and ventroflexion) (Data S1; Figures 3 and S1–S4).

**FIGURE 3:**
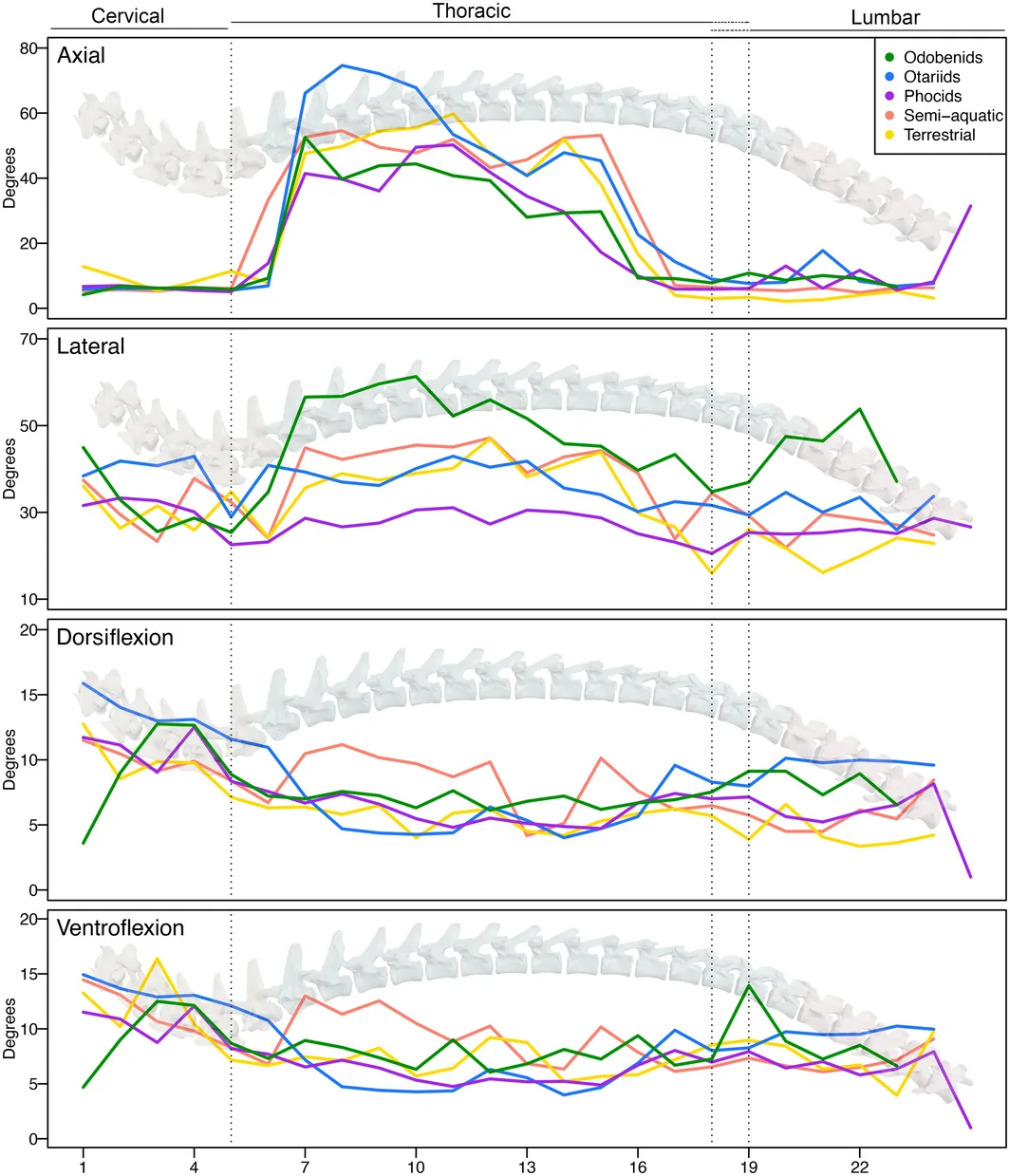
Bivariate graphs of *oROM* values (y‐axis) against intervertebral joints (x‐axis) obtained for each plane. The data represent averages per joint and group (for individual level data, see Figures S1–S4). Vertical dotted lines represent regional boundaries. The thoracolumbar boundary is overlapping because of differences in vertebral formulae across species (see Table S1).

Non‐pinniped taxa exhibit the greatest axial mobility in the cervical region compared to pinnipeds (Figure 3). However, otariids display higher axial mobility in the thoracic series, especially for the anterior ones. Phocids and odobenids exhibit similar *oROM* values of axial mobility, except in the midthoracic region, where phocids show greater axial mobility but less in the anterior and posterior thoracics (Figure 3).

For lateral mobility (Figure 3), non‐pinniped carnivorans exhibit low *oROM* values in the cervical and lumbar series, but similar *oROM* lateral values to otariids and much higher lateral values than phocids in the thoracic region. Among pinnipeds, odobenids demonstrate the highest overall lateral mobility across the entire spine, except in the cervical region, where they show significantly reduced lateral mobility compared to other groups, except for the first joint. Otariids display greater lateral mobility throughout the spine than phocids (Figure 3).

Regarding movement in the dorsiflexion component of the sagittal plane (Figure 3), otariids are characterized by having the highest mobility values across the cervical and lumbar series, followed by odobenids and phocids. In contrast, semi‐aquatic taxa are here characterized by having relatively higher values than terrestrial ones across the thoracic region and most of the lumbar series (Figure 3).

For ventroflexion mobility along joints, otariids stand out for exhibiting greater mobility across the cervical and lumbar series, and, again, semi‐aquatic taxa are characterized by having high mobility, especially in the anterior thoracics (Figure 3).

### 3.2 Correlation analyses among planes of movement

The results of the correlation analyses are shown in Figure 4. In terrestrial carnivorans, the cervical and lumbar regions exhibit largely uncorrelated *oROM* values, with only a significant correlation between axial and lateral motion in the thoracic region. Semi‐aquatic taxa similarly show uncorrelated *oROM* values for both the cervical and lumbar regions, except for a consistent correlation between ventroflexion and dorsiflexion. However, in their thoracic region, *oROM* values are strongly correlated across planes.

**FIGURE 4:**
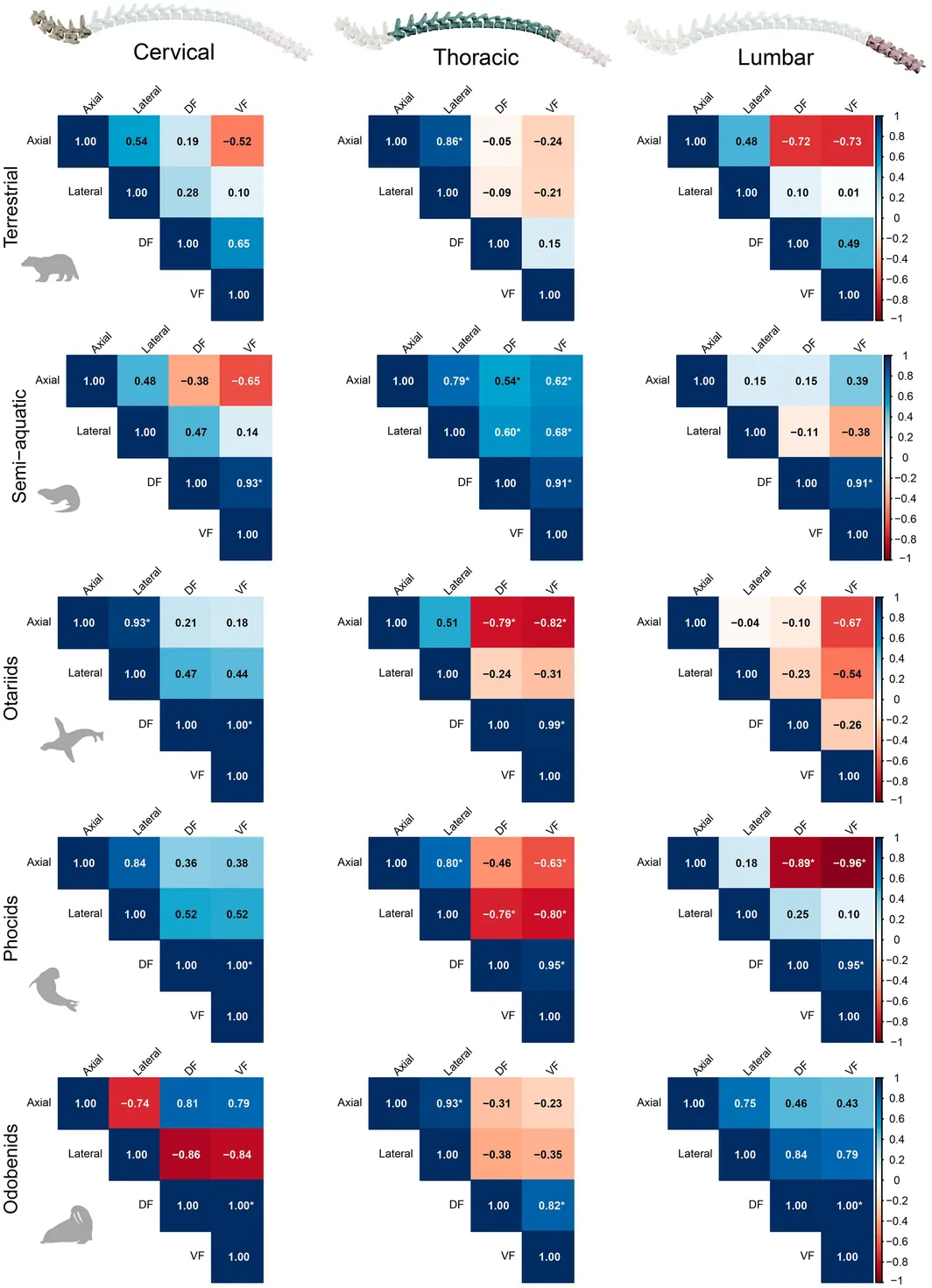
Heatmaps obtained from the correlation analysis of averaged *oROM* values. Data represent averages per joint and group. Asterisks denote significant correlations at the level of 0.05. DF, dorsiflexion; VF, ventroflexion.

In contrast, pinnipeds exhibit increased interplanar coordination of *oROM* values, particularly between ventroflexion and dorsiflexion—except in the lumbar series of otariids. Otariids show a significant correlation between axial and lateral motion in the cervical region. In the thoracic region, axial *oROM* values are significantly correlated with both components of sagittal motion (ventroflexion and dorsiflexion), which are also correlated with each other. Their lumbar region, however, shows no significant correlations between any planes of motion.

Phocids display the highest overall coordination among movement planes within pinnipeds. They show strong correlations between ventroflexion and dorsiflexion in both the cervical and thoracic regions, and additional correlations between axial motion and both components of sagittal motion. Their lumbar region, in particular, exhibits extensive interplanar correlations, except for lateral motion.

In contrast, odobenids show the lowest degree of interplanar coordination among pinnipeds. While the two sagittal components (ventroflexion and dorsiflexion) are significantly correlated across all three regions, only in the thoracic region do axial and lateral motion also exhibit a significant correlation.

### 3.3 Principal components analyses (PCAs) and PERMANOVA tests

In all three regions (Figures 5, 6, 7), the first principal component (PCI) accounted for a range of the original variance between 46% and 54%, while the second component (PCII) explained between 26% and 32% (Table 2). The first PC captured the highest proportion of variance in the thoracic region, followed by the cervical region, and lastly the lumbar one.

**FIGURE 5:**
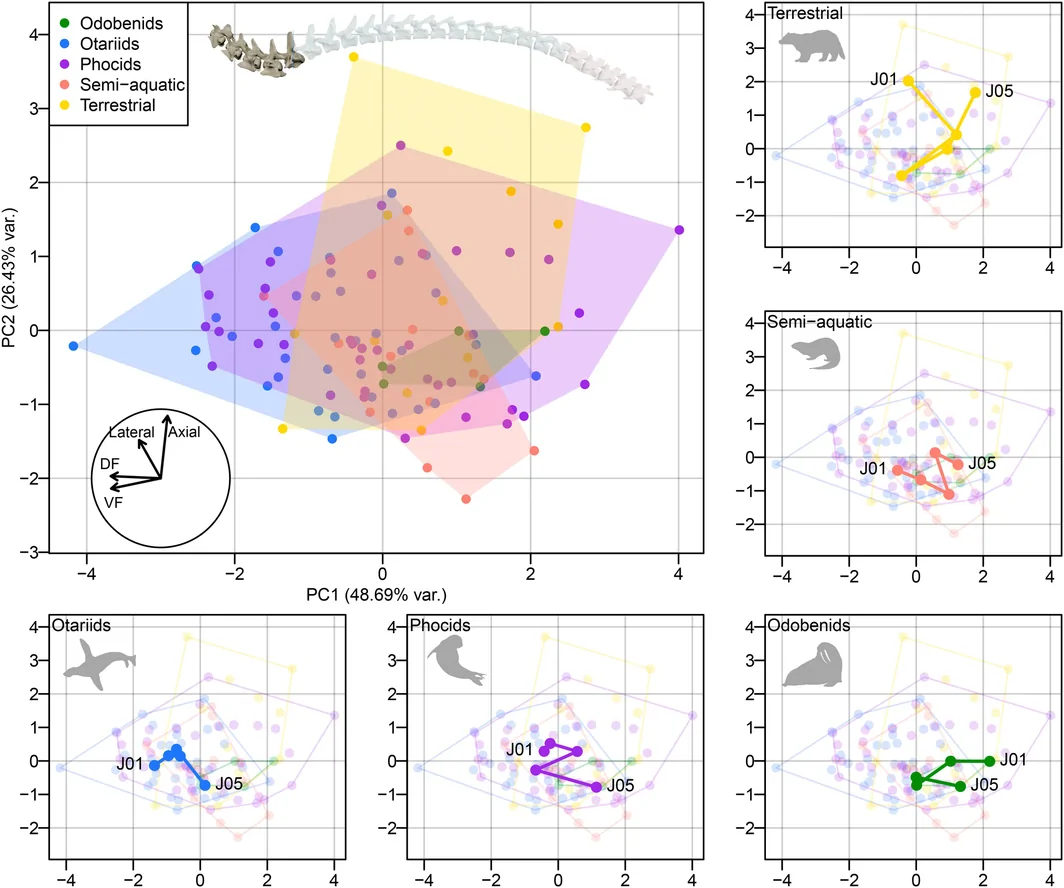
Kinematic space derived from Principal Component Analyses (PCA) from the cervical region. The large panel shows the bivariate plot depicted from the scores of intervertebral joints on the first two eigenvectors obtained from the PCA of *oROM* values obtained from individual‐level data. The small panels show the averages of the scores per joint and per group. DF, dorsiflexion; VF, ventroflexion; J, joint.

**FIGURE 6:**
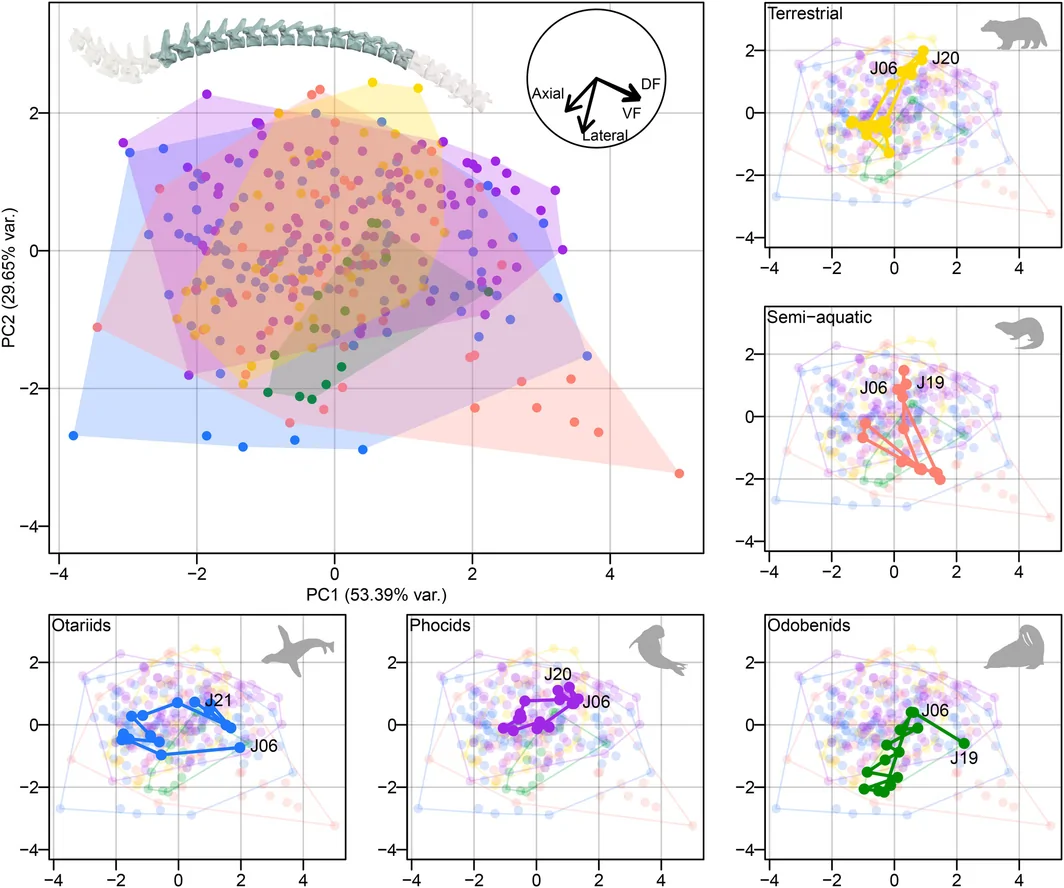
Kinematic space derived from Principal Component Analyses (PCA) from the thoracic region. The large panel shows the bivariate plot depicted from the scores of intervertebral joints on the first two eigenvectors obtained from the PCA of *oROM* values obtained from individual‐level data. The small panels show the averages of the scores per joint and per group. Color coding as in Figure 5. DF, dorsiflexion; VF, ventroflexion; J, joint. Note that in the small panel of terrestrial taxa, J20 represents the *oROM* value of only *Meles meles* because it is the only species with 15 thoracics. The same applies to J21 of otariids, which represents the *oROM* value of *Eumetopias jubatus* because it is the only otariid with 16 thoracics.

**FIGURE 7:**
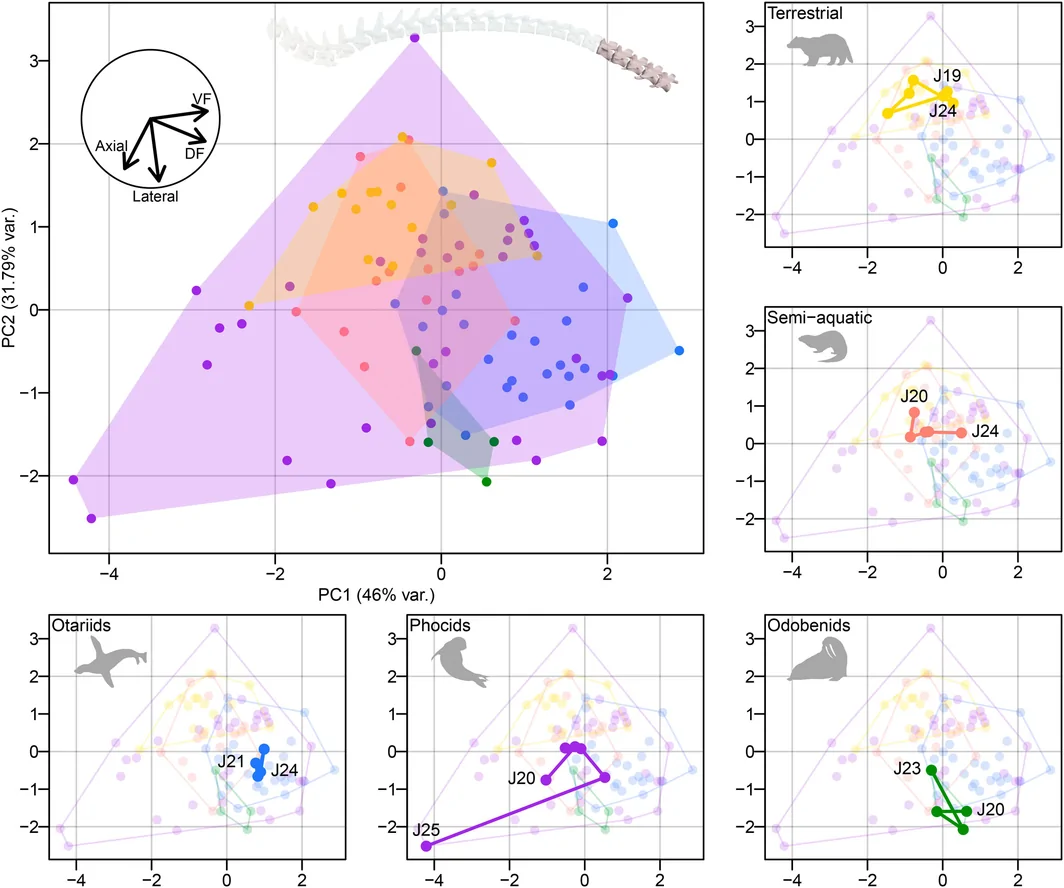
Kinematic space derived from Principal Component Analyses (PCA) from the lumbar region. The large panel shows the bivariate plot depicted from the scores of intervertebral joints on the first two eigenvectors obtained from the PCA of *oROM* values obtained from individual‐level data. The small panels show the averages of the scores per joint and per group. Color coding as in Figure 5. DF, dorsiflexion; VF, ventroflexion; J, joint. Note that in the small panel of terrestrial taxa, J19 represents the *oROM* value of only *Martes foina*, because it is the only species with 13 thoracics. The same applies to J25 of phocids, which represents the *oROM* value of *Mirounga angustirostris* because it is the only phocid with 6 lumbars. Finally, J20 of phocids represents the *oROM* value of only *Hydrurga leptonyx* and *Cystophora crystata*, because they are the only phocids with 14 thoracics.

**TABLE 2:** Eigenvalues and percentage of original variance explained of each eigenvector obtained from the three PCAs computed from *oROM* values.

Eigenvector	Eigenvalue	Variance
C_PC1	1.9476	48.69
C_PC2	1.0571	26.43
T_PC1	2.1355	53.39
T_PC2	1.1859	29.65
Lu_PC1	1.8399	46.00
Lu_PC2	1.2715	31.79

Abbreviations: C, cervical; T, thoracic; Lu, lumbar.

#### 3.3.1 Cervical region

The bivariate plot depicted from the first two eigenvectors obtained in PCA of the cervical region computed from individual‐level data of *oROM* values obtained for each intervertebral joint is represented in Figure 5. PC1 accounts for 48.69% of the variance (Table 2), with negative values primarily associated with greater sagittal mobility (in both ventroflexion and dorsiflexion) and, to a lesser extent, increased lateral movement. Conversely, positive values indicate reduced mobility across all planes (Figure 5). PC2 accounts for 26.43% of the total variance (Table 2), with positive values indicating greater mobility in the axial and lateral axes and negative values representing reduced movement across all planes (Figure 5).

The results of the PERMANOVA test computed to test for differences among groups from the scores taken on the first two principal components of all cervical joints for each species are shown in Table 3. These results indicate that the cervical joints of terrestrial taxa and otariids differ significantly from those of the other groups. Despite this, averaging the scores of each joint across all species within a group reveals broad differences in kinematic space occupation.

**TABLE 3:** Results of the PERMANOVA and *post‐hoc* tests computed from the scores of the first two PCs by region with the taxonomic/locomotory groups (Phocidae, Otariidae, Odobenidae, Semi‐aquatic and Terrestrial) as independent variables.

	Explained	F‐exp	*p*‐value adj
Cervical			
Odobenids	2.876738	1.57076784	0.217
Otariids	8.7468408	4.77598448	0.005*
Phocids	0.1173678	0.06408564	0.916
Semi‐aquatic	4.5953356	2.50916325	0.125
Terrestrial	9.3644573	5.11321789	0.012*
Total	25.7007396	3.50830477	0.003996*
Thoracic			
Odobenids	12.128792	6.593726	0.002*
Otariids	5.407583	2.939792	0.02*
Phocids	18.751273	10.193988	0.002*
Semi‐aquatic	20.047321	10.898574	0.002*
Terrestrial	2.400621	1.305079	0.233
Total	58.735589	7.98279	0.000999*
Lumbar			
Odobenids	6.568375	4.101758	0.028*
Otariids	13.620037	8.505315	0.002*
Phocids	2.345928	1.464963	0.119
Semi‐aquatic	2.991091	1.867849	0.119
Terrestrial	17.156604	10.713798	0.002*
Total	42.682034	6.663421	0.000999*

*Note*: The amount of variance explained (Explained), the probability of F distribution (F‐exp), and the associated adjusted *p*‐value (*p*‐value adj) are shown.

The first PC differentiates among the three groups of pinnipeds, while the second tends to separate non‐pinnipeds (particularly terrestrial taxa) from pinnipeds. According to this, the otariids are characterized by having great sagittal mobility in both ventroflexion and dorsiflexion, followed by phocids and odobenids, which show the least sagittal mobility among pinnipeds, but like terrestrial and semi‐aquatic taxa. In contrast, the average scores of each joint across all species taken on the second PC indicate that terrestrial taxa (particularly the first and last cervical joints) are characterized by having great lateral and axial mobility. In contrast, pinnipeds and semi‐aquatic carnivorans show reduced axial and lateral mobilities across cervical joints.

#### 3.3.2 Thoracic region

The bivariate plot depicted from the first two eigenvectors obtained in PCA of the thoracic region computed from individual‐level data of *oROM* values obtained for each intervertebral joint is represented in Figure 6. The first PC accounts for 53.39% of the total variance, with positive values primarily indicating a greater range of motion in the sagittal plane for both ventroflexion and dorsiflexion, while negative values correspond to joints with limited mobility in the sagittal plane and some movement in the axial plane (Table 2). The second PC explains 29.65% of the variance, where negative values are associated with joints exhibiting greater lateral and axial movement, whereas positive values indicate joints with restricted mobility in these planes (Table 2).

The results of the PERMANOVA tests indicate that the thoracic joints of all groups are statistically different from those of the other groups, excepting those of terrestrial taxa (Table 3).

The shared morphospace with all joints of Figure 6 shows overlap between groups, but the subpanels allow us to identify distinctive internal trajectories within each group, which is key to understanding different patterns of intervertebral joint mobility across groups.

In the thoracic region, the first PC primarily separates otariids from the rest of the groups, as their joints score more negatively on this eigenvector, especially in the posterior thoracic joints (e.g., J15–J18), indicating restricted sagittal movements in both ventroflexion and dorsiflexion and some degree of mobility on the axial plane compared to other taxa. In contrast, terrestrial, semi‐aquatic, phocid, and odobenid taxa tend to score positively on this eigenvector (except for some joints of odobenids), suggesting reduced mobility across all planes.

The second PC separates terrestrial and semi‐aquatic taxa with the most positive scores, especially in the anterior and posterior joints (e.g., J06–J18), suggesting reduced axial and lateral mobilities. Pinniped taxa, especially odobenids, tend to occupy lower regions of PC2, reflecting high axial and lateral mobility, although phocids and otariids are positioned more centrally, consistent with moderate axial and lateral movements along the thoracic region.

#### 3.3.3 Lumbar region

The bivariate plot depicted from the first two eigenvectors obtained in PCA of the lumbar region computed from individual‐level data of *oROM* values obtained for each intervertebral joint is represented in Figure 7.

The first PC explains 46.00% of the total variance. Positive values correspond to greater mobility in ventroflexion and dorsiflexion, whereas negative values indicate reduced movement in these planes and some degree of movement in the axial plane (Table 2). The second PC accounts for 31.79% of the variance. Positive values in this component are associated with reduced mobility in all planes except for ventroflexion, whereas negative values reflect increased mobility in the axial and lateral planes but also in dorsiflexion (Figure 7).

The results of the PERMANOVA tests indicate that the lumbar joints of all groups are statistically different from those of the other groups, excepting the values taken by phocids and semi‐aquatic taxa. Score averages of each joint across all species within a groupreveal broad differences of kinematic space occupation among groups (Table 3).

The first PC differentiates non‐pinniped carnivorans with negative scores from otariids and odobenids, while phocids overlap considerably with the non‐pinniped taxa in this axis. This indicates a greater restriction of lumbar mobility across the sagittal plane for both ventroflexion and dorsiflexion, compared to the higher mobility exhibited by all aquatic carnivorans, which tend to score more positively (i.e., odobenids and otariids) or occupy a more central position (phocids) (Figure 7). It is worth noting that the value of J25 in phocids represents the absolute *oROM* of *Mirounga angustirostris*, the only phocid species in our sample with six lumbar vertebrae, and thus has no direct anatomical equivalent in the other groups. Moreover, *M. angustirostris* exhibits the largest body mass among phocids, which likely accounts for its deviation from the general pattern (Mittermeier & Wilson, 2014).

The second PC also differentiates terrestrial and semi‐aquatic taxa with positive scores from pinnipeds scoring negatively (Figure 7), although semi‐aquatic taxa occupy an intermediate position between terrestrial taxa and pinnipeds. This indicates that the lumbar series of terrestrial taxa, and to a lower degree semi‐aquatic species, present restricted mobilities in the axial and lateral planes. In contrast, all pinnipeds present higher mobility in these planes than non‐pinniped taxa.

## 4 DISCUSSION

### 4.1 The impact of the land‐to‐sea transition in vertebral column mobility

Our results show that, while absolute *oROM* values overlap between groups, the PCA indicates that terrestrial taxa exhibit comparatively lower vertebral mobility in the thoracic and lumbar regions when all motion variables are considered together. This pattern is consistent with the mechanical demands of supporting body weight and maintaining stability during high‐speed locomotion (e.g., galloping) using asymmetrical quadrupedal gaits (Reilly et al., 2007; Schilling & Hackert, 2006), and likely reflects the more constrained axial skeleton of terrestrial carnivores (Figueirido et al., 2021).

Their high cervical mobility may reflect the need for head maneuverability during prey handling and feeding behavior on land (Van Valkenburgh, 1996; Böhmer & Ocak, 2024).

Semi‐aquatic taxa, which retain more terrestrial habits than pinnipeds, show slightly increased vertebral mobility, particularly in the lumbar region, likely due to a partial relaxation of gravitational constraints (Esteban et al., 2023b; Fish, 1996).

In contrast, all pinnipeds show increased intervertebral mobility in the lumbar series, where high values in axial and lateral movement are consistent with an undulatory propulsion in water, as well as with the ability to perform rapid turnings important for maneuverability (Fish et al., 2003; Leahy et al., 2021). In general, pinnipeds exhibit reduced cervical mobility across the axial and lateral planes, which is consistent with an environment where the head rotates more coordinately with the body, as prey handling and manipulation are not as important as in fully terrestrial forms.

Most correlations between movement planes were non‐significant for terrestrial taxa, which reflect a functionally decoupled vertebral column, where different planes of motion can act relatively independently. This decoupling is somewhat coherent with the different roles (weight support and quadrupedal locomotion) of the vertebral column.

In contrast, semiaquatic taxa show a pattern that is somewhat intermediate to the one observed in terrestrial carnivorans and pinnipeds. Some correlations between movement planes become significant, indicating a partial shift toward movement coordination, likely related to the demands of trunk stabilization during swimming. However, their movement remains relatively less coordinated compared to pinniped species, and most interplanar correlations for the cervical and lumbar regions remain absent, which suggests that they retain the pattern of movement coordination observed in terrestrial taxa.

Pinnipeds exhibit a general shift toward coordinated vertebral motion, which suggests that the spine kinematics of pinnipeds is more coordinated than that of non‐pinniped taxa. This may reflect the column‐driven propulsion in pinnipeds, in contrast to the limb‐driven locomotion of terrestrial and semi‐aquatic taxa.

### 4.2 Divergent mobility patterns within pinnipeds relate to their different swimming styles

Although all pinnipeds share adaptations to the marine environment, their vertebral column kinematics show marked differences, which may reflect their distinct locomotor and ecological strategies.

Otariids show high sagittal mobility in both cervical and lumbar regions, while their thoracic region is rigid across all planes except in the lateral one. This probably relates to the demands of pectoral rowing, which requires trunk stabilization for forward thrust, but allows lateral movement when not coordinated with other planes.

Their enhanced cervical mobility may facilitate head maneuverability and agility for prey capture and, therefore, maintain active neck stiffness through muscular control (Keller et al., 2025). This is also supported by the relative kinematic independence of the cervical region, likely reflecting compensatory movements during prey capture or head maneuvers. Moreover, the otariids use “head shaking” at the surface of the water when feeding on large prey (Hocking et al. 2016, 2017; Kienle et al., 2017).

Some degree of lateral and sagittal movements characterizes the lumbar region of otariids, and our correlation analyses indicate an absence of movement coordination across all planes. This kinematic independence of the lumbar region may support rapid and agile turnings while chasing prey, but it could also be consistent with their “quadrupedal” locomotion on land (English, 1976; Kerr et al., 2022; Peterson & Bartholomew, 1967).

Cervical mobility of phocids is reduced compared to otariids. Their stiffer neck probably relates to a different adaptation based in passive resistance to minimize drag in aquatic locomotion (Keller et al., 2025). As otariids, phocids exhibit a gradient in flexibility, but with reduced cervical motion (Berta et al., 2006; Hocking et al., 2016, 2017; Kienle et al., 2017).

Both otariids and phocids show trunk rigidity and increased coordination in the thoracic region. However, phocids exhibit the highest degree of movement coordination among pinnipeds but restricted lateral motion compared to otariids. This rigidity and coordination may be associated with trunk stabilization during swimming by pelvic oscillation (Esteban et al., 2023b; Kuhn & Frey, 2012), a locomotion strategy in which the anterior section of the body remains rigid while the hind flippers exert lateral sweeps complemented by the pelvic girdle (Kuhn & Frey, 2012). Moreover, on land, phocids move via caterpillar‐like undulations, which may further constrain both cervical and thoracic motion. The higher degree of coordination in phocids compared to otariids suggests that trunk stabilization is more important for pelvic oscillation than for pectoral rowing.

Although the separation of phocids in the lumbar region was not statistically significant when using individual‐level data with PERMANOVA tests, their group means shown in the PCA suggest a clear tendency toward greater axial and lateral mobility, reflecting their reliance on pelvic oscillation for propulsion in water. Accordingly, the obtained absence of significance for the lumbar region based on individual‐level data should be due to their high size disparity within the group (from *Phoca* to *Mirounga*) (Mittermeier & Wilson, 2014).

Odobenids exhibit marked rigidity in the cervical region, especially in the axial and lateral planes. This is consistent with their unique feeding ecology, as walruses use their tusks to excavate benthic mollusks, anchoring themselves to the seafloor, a behavior that demands cervical stabilization rather than flexibility (Gordon, 1979). Movement coordination seems not to be restricted in odobenids as there is only one cervical correlation between the two components of sagittal motion.

Odobenids display unexpectedly high thoracic mobility, likely due to their mixed swimming strategy (Gordon, 1981, 1983). In contrast to otariids and phocids, they are not mechanically constrained by a single propulsion mode, which may allow greater flexibility in the thoracic region. These reduced mechanical constraints compared to otariids and phocids may explain the relatively high thoracic mobility of *Odobenus rosmarus*, which may also support their ability to move on land by generating motion mainly with the anterior spine (Gordon, 1981, 1983).

Surprisingly, odobenids show greater lumbar mobility than phocids, despite the latter being strict pelvic oscillators. This suggests that phocids may concentrate movement around the lumbosacral joint, while odobenids engage the entire lumbar region more actively. Indeed, phocids possess a wide sacrum with a large endplate (a layer of cartilage and bone that separates the intervertebral disks from the adjacent vertebrae) (Boszczyk et al., 2001; Esteban et al., 2023b), as well as hypertrophied epaxial muscles (Esteban et al., 2023a; Esteban et al., 2023b), which are related to propulsion during swimming. Odobenids, however, exhibit extreme lateral flexibility across the lumbar series (Esteban et al., 2023b; Gordon, 1981).

### 4.3 Relation of kinematic patterns with vertebral column integration and modularity

Previous study on the evolution of the pinniped vertebral column proposed that there is a link between the degree of morphological integration and vertebral column mobility. Specifically, Martín‐Serra et al. (2021) for terrestrial carnivorans and Esteban et al. (2023a) for pinnipeds hypothesized that high intervertebral integration may constrain joint‐specific motion, whereas reduced integration could permit greater capabilities of column mobility. These hypotheses were solely based on morphometric data. In this study, we provide, for the first time, estimates of intervertebral joint mobility in pinnipeds and some non‐pinniped taxa. This allows us to evaluate whether differences in vertebral integration and modularity correspond with spinal mobility, and whether distinct locomotor strategies among pinnipeds (i.e., pectoral rowing vs. pelvic oscillation) are reflected in different functional organizations of the axial skeleton.

Martín‐Serra et al. (2021) found that terrestrial carnivores have three integrated modules: cervical, anterodorsal (anterior thoracics), and posterodorsal (posterior thoracics plus lumbars). The diaphragmatic vertebra separates the two dorsal modules but is not integrated with either, possibly allowing mobility at the diaphragmatic joint complex (Figueirido et al., 2023; Filler, 1986; Slijper, 1946). Moreover, Martín‐Serra et al. (2021) interpreted that while the anterodorsal module is thought to reflect mechanical constraints imposed by the thoracic rib cage, the posterodorsal module may relate to limiting extension in the posterior back. In addition, this study indicates that diaphragmatic vertebrae, which mark the boundary between both dorsal modules, would facilitate more mobility at the diaphragmatic joint complex (Figueirido et al., 2023; Filler, 1986; Slijper, 1946).

Esteban et al. (2023a) concluded that the vertebral column of all pinnipeds is less integrated than the vertebral column of terrestrial taxa. Our results confirm higher vertebral mobility in pinnipeds compared to terrestrial and semiaquatic taxa, except in the cervical region. Moreover, Esteban et al. (2023a) demonstrated differing patterns of intervertebral integration between phocids and otariids.

Our findings support the hypothesis of Esteban et al. (2023a) that otariids do not show a clear modular pattern and maintain high spinal mobility, consistent with their maneuverability and lack of column‐driven propulsion (Kuhn & Frey, 2012; Pierce et al., 2011). In contrast, phocids have a rigid thoracic region and a decoupled posterior column, which seem to confirm that their highly integrated thoracic region is also motion restricted.

Esteban et al. (2023a) proposed that the diaphragmatic vertebra is less functionally distinct in pinnipeds, being integrated within the thoracic segment, possibly due to the absence of asymmetrical gaits at high speeds (Filler, 1986; Jones et al., 2024; Jones et al., 2021; Reilly et al., 2007; Schilling & Hackert, 2006). Our findings reveal that the diaphragmatic vertebrae in pinnipeds do not exhibit exceptional mobility in any plane (Figure S5), confirming that its critical role as a pivot point between the anterior and posterior vertebrae (Martín‐Serra et al., 2021) of the trunk has been lost.

### 4.4 Study limitations

In this study, we quantified the osteological range of motion (*oROM*) as a proxy for intervertebral joint mobility in the cervical, thoracic, and lumbar regions of five carnivoran groups (terrestrial taxa, semi‐aquatic taxa, phocids, otariids, and odobenids) using virtually assembled columns from 3D models. We used *Autobend* (Jones, Brocklehurst, & Pierce, 2021), an automated tool validated with cadaveric data from a tegu and a cat—species with very different vertebral biomechanics (e.g., sprawling vs. parasagittal posture/locomotion, respectively) and vertebral articulations (i.e., procoelous vs. acoelous vertebrae, respectively), to demonstrate its broad applicability across tetrapods. Following Jones, Brocklehurst, & Pierce (2021), we averaged the results obtained from eight parameter combinations (varying joint spacing, intersection threshold, and tension estimates) to obtain robust estimates of mobility.

Despite this, the ideal scenario would include a sensitivity analysis of parameter variation using pinniped cadaveric data. This would allow biomechanical validation fitted to the specific traits of the pinniped vertebral column. Future studies, including empirical data for aquatic carnivores, would help refine and confirm the findings presented here.

Another caveat is that our models did not include the rib cage, as in the original validation of *AutoBend*. Thus, our thoracic analyses likely overestimate actual mobility due to the absence of rib articulations. This likely explains the higher thoracic *oROM* values relative to the cervical and lumbar regions. However, this bias is consistent across taxa, so interspecific comparisons for the thoracic series remain valid, even if absolute values should be interpreted with caution.

Our sample of terrestrial taxa is limited to musteloids and, therefore, including other carnivoran families could reveal greater variation in *oROM* for terrestrial taxa. Nevertheless, we believe the terrestrial species included here are representative enough for comparison with semi‐aquatic and fully aquatic taxa. Similarly, although the only extant odobenid (*O. rosmarus*) was included in our sample, future studies incorporating fossil species could further refine our results.

## 5 CONCLUSIONS

Our results demonstrate that the land‐to‐sea transition in carnivorans involved a shift in intervertebral joint mobility. Terrestrial taxa show reduced mobility in the thoracic and lumbar regions, but increased cervical mobility, likely linked to prey handling on land. In contrast, pinnipeds exhibit greater vertebral mobility overall, particularly in the lumbar region, probably reflecting their role in propulsion (i.e., phocids) and maneuverability (i.e., otariids) in water. However, pinnipeds show reduced cervical mobility, which likely reflects a combination of reduced need for prey manipulation and adaptations for streamlined swimming.

Semi‐aquatic species show intermediate mobility values, suggesting a partial relaxation of terrestrial constraints and an incipient adaptation to inhabit aquatic environments. Similarly, they exhibit partial movement coordination, particularly in the thoracic region, between the decoupled patterns of terrestrial taxa and the more coordinated motion of pinnipeds. This increased coordination in pinnipeds may reflect the functional demands of column‐driven propulsion.

Pinnipeds have diversified into distinct kinematic regimes associated with their locomotor strategies, pectoral rowing in otariids (Fish, 2016), pelvic oscillation in phocids (Pierce et al., 2011), and mixed fore‐ and hindlimb locomotion in walruses (Gordon, 1981, 1983).

Otariids show high sagittal and lateral mobility in the cervical and lumbar regions, reflecting their pectoral‐rowing swimming style, agility in prey capture, and quasi‐quadrupedal movement on land. Phocids have a rigid, highly coordinated thoracic region but a relatively flexible lumbar spine, allowing lateral motion during swimming with a pelvic‐oscillation strategy. This thoracic rigidity may also explain their limited mobility on land.

Odobenids have a rigid neck adapted for foraging on the seafloor with their tusks in search of marine invertebrates, but also an extremely mobile lumbar region and a relatively flexible thoracic region. This thoracic mobility may relate to the ability of *O. rosmarus* to move on land by placing its forelimbs beneath its body and generating motion with the anterior spine (Gordon, 1981). Although the greater lateral mobility of the lumbar region in odobenids compared to phocids is difficult to explain with the current data, we hypothesize that odobenids involve the entire lumbar series more actively during pelvic oscillation, whereas phocids likely rely mainly on the lumbosacral joint.

Finally, our study indicates a possible association between vertebral mobility and patterns of integration and modularity. Non‐pinniped taxa exhibit higher integration and correspondingly reduced mobility (Esteban et al., 2023a; Figueirido et al., 2021; Martín‐Serra et al., 2021). Within pinnipeds, phocids possess a single, highly integrated thoracic module (Esteban et al., 2023a), which is also the most motion‐restricted region in our dataset. In contrast, otariids lack a strong modular organization and exhibit generally higher mobility, particularly in the thoracic region, consistent with their need for rapid turnings and maneuverability.

## AUTHOR CONTRIBUTIONS

**Juan Miguel Esteban:** Writing – original draft; investigation; methodology; data curation; formal analysis; writing – review and editing; visualization; validation. **Alberto Martín‐Serra:** Investigation; methodology; validation; visualization; writing – review and editing. **Alejandro Pérez‐Ramos:** Investigation; data curation; methodology; writting – review and editing. **Borja Figueirido:** Conceptualization; investigation; funding acquisition; writing – original draft; formal analysis; project administration; supervision; data curation; writing – review and editing; visualization.

## FUNDING INFORMATION

This study has been funded by the Spanish Ministry of Science and Universities (grant numbers PID2019‐111185GB‐I00) and Junta de Andalucía (P18‐FR‐3193) to Borja Figueirido. Funding for open access charge was provided by Universidad de Malaga/CBUA.

## CONFLICT OF INTEREST STATEMENT

The authors declare no conflict of interest.

## Supporting information

Supporting Information.

Supporting Information.

Supporting Information.

## Data Availability

The data that supports the findings of this study are available in the supplementary material of this article.
