# Supplementary material for: Patterns of spinal motion, kinematic spaces and the land‐to‐sea transition in carnivorans
Source: Anat Rec (Hoboken). 2025 Dec 24;309(11):3257–75. doi: 10.1002/ar.70102 (PMC13645033; doi:10.1002/ar.70102)
Supplement: Supplementary file 2 [file AR-309-3257-s003.docx]

**SUPPLEMENTARY FIGURES**

| **Name** | **C** | **T** | **L** | **DP** | **CJ** | **TJ** | **LJ** | **DJ** |
| --- | --- | --- | --- | --- | --- | --- | --- | --- |
| *E. lutris* | 5 | 14 | 6 | T12 | J1-J5 | J6-J19 | J20-J24 | J17 |
| *L. canadensis* | 5 | 14 | 6 | T12 | J1-J5 | J6-J19 | J20-J24 | J17 |
| *L. lutra* | 5 | 14 | 6 | T12 | J1-J5 | J6-J19 | J20-J24 | J17 |
| *M. foina* | 5 | 13 | 6 | T11 | J1-J5 | J6-J18 | J19-J23 | J16 |
| *M. meles* | 5 | 15 | 5 | T12 | J1-J5 | J6-J20 | J21-J24 | J17 |
| *Procyon lotor* | 5 | 14 | 6 | T11 | J1-J5 | J6-J19 | J20-J24 | J16 |
| *Z. californianus* | 5 | 15 | 5 | T11 | J1-J5 | J6-J20 | J21-J24 | J16 |
| *E. jubatus* | 5 | 16 | 4 | T12 | J1-J5 | J6-J21 | J22-J24 | J17 |
| *N. cinerea* | 5 | 15 | 5 | T11 | J1-J5 | J6-J20 | J21-J24 | J16 |
| *O. byronia* | 5 | 15 | 5 | T12 | J1-J5 | J6-J20 | J21-J24 | J17 |
| *C. ursinus* | 5 | 15 | 5 | T11 | J1-J5 | J6-J20 | J21-J24 | J16 |
| *Z. wollebaeki* | 5 | 15 | 5 | T12 | J1-J5 | J6-J20 | J21-J24 | J17 |
| *A. pusillus* | 5 | 15 | 5 | T12 | J1-J5 | J6-J20 | J21-J24 | J17 |
| *C. cristata* | 5 | 14 | 5 | T10 | J1-J5 | J6-J19 | J20-J23 | J15 |
| *M. tropicalis* | 5 | 15 | 5 | T11 | J1-J5 | J6-J20 | J21-J24 | J16 |
| *H. grypus* | 5 | 15 | 5 | T11 | J1-J5 | J6-J20 | J21-J24 | J16 |
| *M. angustirostris* | 5 | 15 | 6 | T12 | J1-J5 | J6-J20 | J21-J25 | J17 |
| *H. leptonyx* | 5 | 14 | 6 | T09 | J1-J5 | J6-J19 | J20-J24 | J14 |
| *P. groenlandicus* | 5 | 15 | 5 | T10 | J1-J5 | J6-J20 | J21-J24 | J15 |
| *P. vitulina* | 5 | 15 | 5 | T10 | J1-J5 | J6-J20 | J21-J24 | J15 |
| *E. barbatus* | 5 | 15 | 5 | T11 | J1-J5 | J6-J20 | J21-J24 | J16 |
| *O. rosmarus* | 5 | 14 | 5 | T11 | J1-J5 | J6-J19 | J20-J23 | J16 |

Figure S1.Bivariate graphs of oROM values against intervertebral joint for the axial plane. Individual-level data is shown for all groups. Dotted lines indicate regions (from left to right: cervical, thoracic and lumbar).

Figure S2.Bivariate graphs of oROM values against intervertebral joint for the lateral plane. Individual-level data is shown for all groups. Dotted lines indicate regions (from left to right: cervical, thoracic and lumbar).

Figure S3.Bivariate graphs of oROM values against intervertebral joint dorsiflexion. Individual-level data is shown for all groups. Dotted lines indicate regions (from left to right: cervical, thoracic and lumbar).

Figure S4.Bivariate graphs of oROM values against intervertebral joint for ventroflexion. Individual-level data is shown for all groups. Dotted lines indicate regions (from left to right: cervical, thoracic and lumbar).

Figure S5. Box-plots with individual-level values of oROM data obtained for all thoracic joints across the three planes. Asterisks denote diaphragmatic vertebrae. The y-axis are in degrees.
